# Alcohol and cannabis use among adolescents in Flemish secondary school in Brussels: effects of type of education

**DOI:** 10.1186/1471-2458-12-215

**Published:** 2012-03-20

**Authors:** Hans Berten, Dries Cardoen, Ruben Brondeel, Nicole Vettenburg

**Affiliations:** 1 Department of Social Welfare Studies, H. Dunantlaan 2, 9000 Ghent, Belgium; 2 Department of Experimental Clinical and Health Psychology, H. Dunantlaan 2, 9000 Ghent, Belgium

## Abstract

**Background:**

Research regarding socio-economic differences in alcohol and drug use in adolescence yields mixed results. This study hypothesizes that (1) when using education type as a proxy of one's social status, clear differences will exist between students from different types of education, regardless of students' familial socio-economic background; (2) and that the effects of education type differ according to their cultural background.

**Methods:**

Data from the Brussels youth monitor were used, a school survey administered among 1,488 adolescents from the 3rd to 6th year of Flemish secondary education. Data were analyzed using multilevel logistic regression models.

**Results:**

Controlling for their familial background, the results show that native students in lower educational tracks use alcohol and cannabis more often than students in upper educational tracks. Such a relationship was not found for students from another ethnic background.

**Conclusion:**

Results from this study indicate that research into health risks should take into account both adolescents' familial background and individual social position as different components of youngsters' socio-economic background.

## Background

Despite recent signs of a decline in the prevalence of legal and illegal drugs, the use of alcohol and cannabis remains widespread among adolescents and young adults in contemporary European society [1,2]. Many people initiate alcohol and drug use during their years as a teenager [3,4]. According to a recent research report, 75.4% of the Flemish scholars (12-18 years) have drunk alcohol at least once during their lifetime [5]. A vast majority of these respondents (63.4%) even used alcohol during the past 12 months, of which 22% drank alcohol on a regular basis, that is, more than once a week. The same research reported a last year prevalence rate of cannabis among Flemish students of 11.7% of which 2.7% used it on a weekly basis. Approximately one out of five students had smoked cannabis once during their lifetime [5]. This substance use is however not without harm. Many studies acknowledge the acute and longer range health implications of this behavior, both on a personal and societal level [6,7]. Conceivable short-term consequences of adolescent's substance (mis)use are an increased risk of accidental injury and death, of engaging in criminal and delinquent behavior, violence victimization, engaging in unsafe sexual practices, educational failure and depression and suicidal ideation. Beyond these immediate "threats", early alcohol and cannabis consumption is frequently associated with a heightened chance of developing substance use and dependence disorders, major depressive symptoms and other undesirable health outcomes in later adulthood.

Notwithstanding these health implications, for most teenagers it holds that adolescence is a phase of experimentation in the first place. A striking observation is that age specific rates of alcohol and drug use, and related to this conformity to peer pressure and fear of peer rejection, peak in adolescence and drop sharply when entering adulthood [8-10]. Nevertheless, given the possible health consequences, the (ab)use of alcohol and cannabis remains a serious public health concern and considerable and continuing efforts need to be conducted to develop effective interventions in this domain. Therefore, insight in risk factors that constitute teenage substance use is imperative.

Several studies have shown that in adulthood socio-economic differences in alcohol and drug use are relatively consistent [11-13]. People from higher socio-economic strata tend to drink more often but in smaller quantities, while their counterparts in lower socio-economic strata tend to consume alcohol less often but in larger quantities and in a more problematic way. While the existence of class-based differences in alcohol and cannabis use among adults is well established, socio-economic differences in adolescents' substance use are far less investigated, and consistency in present evidence is lacking. According to some research reports, the association between socio-economic status (SES) and substance use in adolescence is similar to patterns found in other life stages, where a lower SES is associated with increased incidence rates of alcohol and cannabis consumption [13-16]. On the other hand, several studies couldn't corroborate these findings, reporting an insignificant, diminished or reversed relationship between a teenagers socio-economic position and his/her alcohol and cannabis use [17-20]. As a possible explanation for these inconsistencies, West and others [21,22] referred to the occurrence of "a process of equalization", in which a transition is taking place from health inequality in childhood to relative equality during adolescence. According to these authors, this "process of equalization" is rooted in the defining characteristics of adolescence as a transitional period (i.e. the growing importance of peers, school environment and youth culture), by which the influence of familial background gets sharply curtailed, resulting in a homogenizing effect [21,22].

However, it can be questioned whether this equalization process is a real-existing phenomenon or rather should be interpreted as an artifact, reflecting the way in which an adolescents' socio-economic position is traditionally measured, that is, using information about the parents' socio-economic status (i.e. parental educational level, parental occupational level and family income) [23-25]. As youngsters strive to obtain more autonomy from their parents and develop their own identity, their social position gets increasingly determined by their own choices and life course plans [24,26-28]. Consequently, the use of merely parental SES markers as indicators of social status during adolescence may not be sufficient [27,29,30]. Following Bourdieu and Passeron [31] and others [24,26] we state that it may be more appropriate to use information about adolescents' educational level, as an indicator of their current individual social position, since this determines to a large extent ones future social class group.

According to Bourdieu and Passeron [31] the educational system plays a fundamental role in the reproduction of social inequalities. This reproduction works through a combination of selection and socialization processes. Selection refers to the differential validation of cultural capital in schools: students that possess the "right" (i.e. dominant middle class) cultural capital have greater chances for academic success, while other students flounder at lower levels of education. That way, scholars from lower social classes tend to concentrate in the lower status education types [32-35]. However, schools also socialize students into particular cultures: higher status education types socialize students towards the dominant middle class cultures, while lower status education types socialize towards lower class cultures [31,34,35]. Hence, the existing social order is maintained and social inequalities are even reinforced. Considering people's health behavior, the awareness of such a process of reproduction calls the question whether social gradients in alcohol and cannabis use merely are the result of differences in students' familial background or whether schools reinforce these inequalities.

In Flanders, the educational level of a student can easily be assessed by the type of education the student follows. The Flemish secondary school system is highly tracked and mainly consists of three different types of education that can be ranked in difficulty level from vocational, technical, to general secondary education. General education is a type of education that provides students with a firm theoretic knowledge foundation for going into higher education. Technical education is both practice- and theory-orientated, so that students can either enter the job market directly or continue their studies in higher education. Vocational education is a mere practical type of education and prepares students to enter the job market directly.

Unlike the existing literature on socio-economic differences, this research was conducted within a multicultural environment, i.e. Belgium's capital region. The particular nature and assembly of this urban region shapes a unique context for this study, since it additionally allows us to examine whether the impact of an adolescents' socio-economic position, as determined by both familial background and education type, on his/her consumption pattern, interacts with ones' cultural origin. Brussels is characterized by a large degree of ethnic diversity, clearly reflected in the composition of the students' population in Dutch-speaking secondary education. Hence, migrant students make up about 60% of the secondary school student body. Furthermore, previously conducted analyses [see [36]] illustrated that 75% of this migrant group are Muslims. Since Muslims are overrepresented within the migrant population and due to the fact that Islamic cultures religiously and often legally prohibit the use of alcohol and cannabis [37,38], one can expect that SES differences in substance use are much more clear for native than for migrant students. As such, their religion may act as a buffer for the effect of education type on risk behaviors such as drinking alcohol or using cannabis.

In summary, the present study contributes to the literature by elucidating the role of education type in students' alcohol and cannabis use, controlling for parental SES markers (i.e. parents educational level and work status). The aim of the study is twofold. First, in line with Bourdieu and Passeron [31], we expect that differences in students' consumption pattern not merely reflect the different backgrounds of these students, but that the school environment contributes something unique. Second, given the strong representation of migrants from Islamic countries in Brussels' schools, and given the enforcing rule of abstinence that is dictated in Islamic culture, we expect education type to have a an effect on native students' substance use in the first place.

## Methods

### Data

The data used in this study were derived from the 'JOP-monitor Brussels'. This is a self-report school survey administered by the Youth Research Platform in 2009-2010. The data consists of a sample (N = 2,513) of 12-20 year old students in all grades (i.e. 1st to 6th year) of Flemish secondary education in Brussels. All Dutch-speaking secondary schools (N = 42) in the Brussels region were repeatedly invited to participate: 32 schools (76.2%) agreed to participate, a number that is quite high for this kind of surveys. The reason why the remaining ten schools did not participate is due to the fact that Flemish schools are commonly swamped with survey requests from researchers, generally resulting in a 'first come, first served' outcome. In each of the participating schools, classes were selected randomly based on study year and type of education. This in order to achieve a balanced representation of the Brussels student population. After being informed about the purpose and the voluntary nature of their participation, 88,6% of the students in these 32 schools (N = 2,513) actually filled in the questionnaire. The remaining 11.4% could not participate due to absence caused by illness or class excursions. The questionnaire was administrated in the presence of a researcher and a teacher during regular class periods. The data collection was approved by the Ethics Committee of the Faculty of Psychology and Educational Sciences of Ghent University.

Since the subdivision between the previously described education types only exists from the 3rd year onwards, we selected respondents in the 3rd to 6th year only, resulting in a subsample of 1,488 students (i.e. 61.8% of the total sample). Despite the high representativeness of the data on both the school and class level [39] this study suffered, like most public survey data, of a substantial percentage of missing values. The variables responsible for most missing observations were the outcome variables (all around 27%), parental work status (22.7%), and parents' educational level (23.6%). The 'mi' package in R developed by Gelman et al. [40] for multiple imputation was used to deal with the missings in the model.

### Measures

In this paper we measured last month incidence rates of (1) beer/wine, (2) spirits and (3) cannabis by using a dichotomized scale (0) 'abstainers and moderate users' versus (1) 'frequent users'. 'Frequent users' are those adolescents who used respectively beers/wines, spirits or cannabis more than three times in the last month.

Familial socio-economic background was operationalized by using information about the educational and occupational level of students' parents. Regarding the parents' occupational status we distinguished between three categories: 1) families where both parents are unemployed; 2) families where only one parent is employed and 3) families where both parents are employed. Likewise, the parents' educational level consists of three categories: 1) 'low-educated families' where none of the parents obtained an university degree or equivalent, 2) 'middle-educated families' where only one parent has a university degree or equivalent, and 3)'high-educated families' where both parents have a university degree or equivalent.

Education type in secondary school consists of three main categories: general education, technical education, and vocational education. Control variables included gender (male vs. female), age and cultural background (native vs. migrant students). 'Native students' are those who have the Belgian nationality, speak French or Dutch with at least one of their parents and have at least one parent of Belgian origin.

## Results

### Education type and student characteristics

The results in Table 1 illustrate that the inflow of students in the different education types is strongly determined by their socio-economic background. Considering the two extremes within education type, it becomes clear that 51.7% of the students in general education grow up in a two income-family. In vocational education this is the case for only 32.1% of the students. In contrast, we counted approximately twice as much unemployed households in vocational education (24.4%) as compared to general education (12.9%). That students' education type can't be isolated from their familial background, becomes even more apparent if we regard differences in parents' educational level. The proportion of students living in 'high-educated families' is significantly higher in general education (32.4%) than in technical (17.1%) and vocational education (13.3%). For migrant students, we observed a similar trend: vocational education included the highest percentage of migrant students (77.4%) followed by technical (57.4%) and general education (52.2%). Gender differences within various education types were not significant.

**Table 1:** Outcome and control variables by type of education

	Education type				
Percentages	general	technical	vocational	Total	***p *(*X***^**2**^)
*More than 3 times in the last month*					
*beer/wine*					n.s.
no	79.3	80.3	82.4	80.3	
yes	20.7	19.7	17.6	19.7	
*strong spirits*					***
no	92.1	88.7	84.9	89.6	
yes	7.9	11.3	15.1	10.4	
*cannabis*					***
no	95.8	92.8	88.6	93.3	
yes	4.2	7.2	11.4	6.7	
*Gender*					n.s.
girls	52.8	55.6	53.0	53.7	
*boys*	47.2	44.4	47.0	46.3	
*Cultural background*					***
natives	47.8	42.6	22.6	40.2	
migrants	52.2	57.4	77.4	59.8	
*Parents' educational level*					***
no parents with higher education	38.5	54.9	67.5	49.4	
one parent with higher education	29.0	28.0	19.3	26.7	
two parents with higher education	32.4	17.1	13.3	23.9	
*Parents' work status*					***
no parents working	12.9	11.5	24.4	14.8	
one parent working	35.5	44.3	43.5	39.7	
two parents working	51.7	44.3	32.1	45.5	

* *p *< 0.05, ** *p *< 0.01, *** *p *< 0.001

### Education type and substance abuse

In general, a fifth of all respondents drunk beer or wine on more than three occasions during the 30 days prior to the survey. This is roughly twice the number of students who frequently (i.e. more than 3 times) used spirits (10.4%) and thrice the number of frequent cannabis users (6.7%). Regarding the association between substance use and education type, a significant association was found for spirits and cannabis. Hence, the proportion of students who used these substances was significantly higher in vocational education, respectively 15.1% and 11.4%, than in technical education (respectively 11.3% and 7.2%). General education counts the lowest percentage of frequent strong spirit (7.9%) and cannabis (4.2%) users. These findings are in line with the raised expectation that frequent substance use is more concentrated among students in lower educational tracks.

### Multilevel analysis

Because cluster sampling methods were used to collect the data and since the dependent variables are dichotomous, multilevel logistic regression techniques were used for the multivariate analysis, with the schools as the higher level units of analysis. The models were fitted in each multiple imputed dataset. The obtained coefficients and standard errors were then pooled according to Rubin's rules [41]. Finally, regular Wald tests are used to evaluate the null-hypotheses that the respective coefficients are equal to zero in the population.

In Table 2, results are shown for the analyses on the full sample of 3 rd to 6th year students. Concerning the sociodemographic factors, gender, age and cultural background, were significantly associated with frequent alcohol and cannabis use. As expected, boys and older students and native students use alcohol and cannabis more frequently. As we hypothesized, our analyses display a clear effect of education type on students' consumption pattern, and these effects prove robust for differences in socio-economic background. Remember however that further analyses should indicate whether these effects are equally strong in both samples of native and migrant teens.

**Table 2:** Multilevel logistic regression for alcohol and cannabis use: results for the full sample

	Beer and wine		Spirits		Cannabis	
	(n = 1,448)					
	OR(95% CI)	**Sig**.	OR(95% CI)	**Sig**.	OR(95% CI)	**Sig**.
*Control variables*						
girls	0.54 (0.36-0.79)	***	0.50 (0.32-0.78)	**	0.29 (0.17-0.49)	***
migrants	0.20 (0.13-0.30)	***	0.41 (0.25-0.69)	***	0.38 (0.19-0.75)	**
age	1.41 (1.24-1.60)	***	1.41 (1.21-1.64)	***	1.38 (1.17-1.62)	***
*Family SES*						
parent's work status						
two parents working	1.49 (0.96-2.30)		1.29 (0.74-2.24)		0.96 (0.51-1.79)	
one parent working	0.70 (0.35-1.40)		1.37 (0.67-2.80)		1.31 (0.58-2.95)	
parents' educational level						
two parents higher education	2.57 (1.48-4.48)	***	2.92 (1.65-5.16)	***	1.98 (0.84-4.66)	
one parent higher education	1.64 (0.96-2.79)		1.33 (0.73-2.42)		1.58 (0.84-2.97)	
*Education type*						
technical	1.23 (0.75-2.01)		1.84 (1.07-3.17)	*	1.71 (0.84-3.47)	
vocational	1.75 (1.01-3.01)	*	3.22 (1.77-5.86)	***	4.04 (1.90-8.61)	***

* *p *< 0.05, ** *p *< 0.01, *** *p *< 0.001

Controlling for all other variables in the model, vocational students were significantly more likely than their counterparts in general education to use spirits or cannabis on a regular basis (OR = 3.22 and OR = 4.04 respectively). In addition, vocational students were also inclined to drink beers and wines more frequently, although the level of statistical significance was rather low here (OR = 1.75). With general education as a reference group, no substantial differences were observed for students in technical education. With regard to students' familial social position, significant effects were found only for parents' educational level. Frequent alcohol users are more often found among children of high than low educated parents. Such a finding was however not found for cannabis use. The parents' work status has no effect on adolescents' alcohol and drug use.

Table 3 and Table 4 repeats the same analyses for native and migrant group students separately. This allows us to examine whether the effects of education type differ according to one's ethnic or cultural background. In accordance with Table 2, both native and migrant students' family background proved to be of limited importance. While effects of parents' work status were absent, significant effects were again found for parents' educational level. Regardless of a students' ethnic/cultural background, growing up in a 'high educated family' increased the risk of frequent alcohol use. The findings in Table 3 further show that, for native Belgians, education type clearly structures their alcohol (spirits) and cannabis use (OR = 4.63 and OR = 6.44 respectively). These differences are again most pronounced between students in general education and students in vocational education. However, also students in technical education drink significantly more spirits than their counterparts in the highest track. No differences between educational tracks were observed for drinking beers and wines. The results in Table 4 also show that, for students from another ethnic/cultural background, no significant differences were found between educational tracks. The only exception is for cannabis use, where we found that migrant students in vocational education use cannabis more often than their counterparts in general education.

**Table 3:** Multilevel logistic regression for alcohol and cannabis use: results for majority students

	Beer and wine		Spirits		Cannabis	
	(n = 594)					
	OR(95% CI)	**Sig**.	OR(95% CI)	**Sig**.	OR(95% CI)	**Sig**.
*Control variables*						
girls	0.49 (0.30-0.81)	**	0.54 (0.27-1.11)		0.37 (0.16-0.81)	*
age	1.44 (1.22-1.69)	***	1.42 (1.14-1.77)	**	1.82 (1.36-2.43)	***
*Family SES*						
parent's work status						
two parents working	1.26 (0.75-2.09)		0.93 (0.46-1.89)		0.96 (0.41-2.26)	
one parent working	0.36 (0.09-1.40)		0.65 (0.12-3.50)		0.50 (0.05-4.93)	
parents' educational level						
two parents higher education	2.35 (1.27-4.38)	**	2.93 (1.24-6.93)	*	2.36 (0.86-6.47)	
one parent higher education	1.60 (0.90-2.85)		1.25 (0.53-2.97)		1.82 (0.70-4.77)	
*Education type*						
technical	1.31 (0.68-2.54)		2.93 (1.31-6.56)	**	1.55 (0.58-4.15)	
vocational	2.13 (1.00-4.53)		4.63 (1.81-11.86)	**	6.44 (2.23-18.55)	***

* *p *< 0.05, ** *p *< 0.01, *** *p *< 0.001

**Table 4:** Multilevel logistic regression for alcohol and cannabis use: results for minority students

	Beer and wine		Spirits		Cannabis	
	(n = 894)					
	OR (95% CI)	**Sig**.	OR (95% CI)	**Sig**.	OR (95% CI)	**Sig**.
*Control variables*						
girls	0.59 (0.29-1.21)		0.40 (0.18-0.88)	*	0.21 (0.08-0.54)	**
age	1.41 (1.14-1.75)	**	1.44 (1.16-1.78)	**	1.17 (0.92-1.48)	
*Family SES*						
parent's work status						
two parents working	2.14 (0.88-5.23)		1.90 (0.74-4.89)		0.81 (0.25-2.59)	
one parent working	1.09 (0.43-2.77)		1.90 (0.75-4.82)		1.56 (0.62-3.91)	
parents' educational level						
two parents higher education	3.21 (1.20-8.59)	*	3.01 (1.10-8.21)	*	1.96 (0.47-8.14)	
one parent higher education	1.65 (0.68-4.04)		1.40 (0.58-3.40)		1.42 (0.52-3.87)	
*Education type*						
technical	1.16 (0.50-2.70)		0.93 (0.37-2.34)		1.35 (0.49-3.69)	
vocational	1.49 (0.65-3.45)		2.08 (0.95-4.57)		2.74 (1.14-6.59)	*

* *p *< 0.05, ** *p *< 0.01, *** *p *< 0.001

## Discussion

Starting from the debate on whether a process of equalization in young people's health is taking place, we posited that in adolescence a shift is taking place away from one's familial background to a social position that is increasingly determined by students' own educational career. In the present study we focused on whether alcohol and drug use differs between students from different educational tracks, independent of their familial socio-economic background. Our results clearly confirmed this hypothesis. Students in lower educational tracks use alcohol and cannabis more often than students in upper educational tracks, although this finding held only for native students. We also illustrated that the effects of education type are not reducible to differences in students' familial background characteristics. These findings are in line with the literature on the role of the educational system in the reproduction of inequalities (Bourdieu and Passeron, 1977). Schools socialize students into particular youth cultures. Although some of these socialization is intended and part of the school culture, much of it is an unintended consequence of the interactions among students and between students and staff.

A question that arises immediately when interpreting these findings is which processes within these schools can explain the link between tracking and substance use. Here, research has shown that the specific subcultures that arise in lower educational strata are characterized by a culture of demotion, feelings of held back with bleak future perspectives, feelings of futility, frustration and strain, or eroded self-esteem [42-45]. Furthermore, teachers in lower tracks have much less expectations from their students (i.e. Pygmalion effect) and also leadership styles clearly differ between education types [46]. All these experiences may push these students towards delinquency or substance use, not only to achieve alternative sources of status in the peer group, but also to handle the strain caused by their negative experiences in these lower educational tracks.

The multicultural character of our study sample additionally allowed us to explore whether the effects of education type differed according to students' ethnic or cultural background. As we expected, effects of education type were observed in the sample of native students, while these effects were absent or negligible in the sample of migrant students. We explained this finding by referring to the buffering effect of religion in the relationship between education type and substance use. Students from Islamic background make out the majority in the group of students from another ethnic background, and these students not only profess their religion in a much more conscious way, they are also the product of families and cultures that put more emphasis on religious values such as for instance abstinence than their native Belgian counterparts [47].

A somewhat remarkable finding of our study was the positive association between parents' educational level and alcohol use. Students from families where parents are highly educated used more alcohol in the last month, independent of the students' educational level. Such a finding was not found for cannabis use. A possible explanation may be that students from high SES backgrounds simply have a more attractive financial background, allowing these youngsters to buy alcohol-related products [20,48-50]. For cannabis on the other hand, the financial strength of students' background may be of minor importance. For instance, cannabis is often consumed and shared in larger groups of teens, and especially the amount of cannabis needed to get high is much cheaper in price than the amount of alcohol needed to get drunk. Furthermore, cannabis is legally prohibited, and is dependent on having the 'right' connections or resources to buy these products, and these connections may be much less dependent on the familial SES background of these students. Other explanations are however possible and further research is necessarily to explain these results.

This study suffered from a few limitations. A first limitation is that this study is based on self-reports, making it plausible that the these self-reports are biased by social desirability pressures. A second limitation relates to the recall period in the study (i.e. incidence rates of alcohol and cannabis in the last month). Since students may experience difficulties in remembering exactly how many times they used these substances during the last 30 days, it may be more accurate to focus on shorter recall periods. Also, the analyses presented here are based on outcomes that measure whether the adolescents consumed alcohol or drugs more than three times in the last month, and thus problematic drinking behavior is not really measured. Thus, it may be that the differences between the educational tracks, but also between the migrant and native groups, become even more pronounced when using measures of binge drinking, alcohol intoxication, etc. Because of the restrictions of our alcohol outcomes, our study did not let us to conclude that the so typical 'reversed pattern' -moderate use of alcohol in higher socio-economic groups, less frequent but more problematic alcohol use in lower socio-economic groups- is also observed in adolescence. However, the literature indicates that once teenagers grow older and enter adulthood, such a pattern may start to unfold [11-13].

## Conclusion

Consistent with equalization theory, this study found little effects of parental SES markers on students' substance use, and where such effects existed they marked social gradients in a reversed pattern. Following West [21,22], adolescence is characterized by a "process of equalization", but only for what concerns their familial socio-economic background. Our study showed, however, that when using education type as a proxy of one's individual socio-economic status, clear differences persist among native Belgians in different educational tracks. Thus, structural causes of inequalities are still prevalent, but they work via the schools' students are ascribed to. For future research this means that it may no longer be sufficient to determine adolescents' social position merely on parental SES indicators. Rather, it is required to chart students' own social position as well, for which education type can be used as a proxy. Otherwise, researchers are blind for the diversity and SES based social stratification that still persists within Belgian, but also other European, schools.

## Competing interests

The authors declare that they have no competing interests.

## Authors' contributions

HB conducted all statistical analyses, wrote, and coordinated the paper. DC co-wrote the manuscript, RB provided statistical support, and NC was co-responsible for the data collection and supervision of the Brussels youth monitor and provided theoretical additions and other comments to the manuscript. All authors commented on drafts, have read the revised manuscript, and have approved the final version.

## Pre-publication history

The pre-publication history for this paper can be accessed here:

http://www.biomedcentral.com/1471-2458/12/215/prepub
